# Physician and Pharmacist Liability: Medicolegal Cases That are Tough Pills to Swallow

**DOI:** 10.5811/cpcem.2021.4.51851

**Published:** 2021-05-10

**Authors:** John C.S. Schupbach, Maria C. Kaisler, Gregory P. Moore, Benjamin J. Sandefur

**Affiliations:** Mayo Clinic, Department of Emergency Medicine, Rochester, Minnesota

**Keywords:** Malpractice, medication error, prescribing, liability

## Abstract

We present four medicolegal cases involving medication errors, which led to patient harm and subsequent settlements or jury awards to patients. These cases each involved scenarios in which a medication was inappropriately prescribed and/or inappropriately dispensed. In such cases, it is often not obvious whether the physician or pharmacist is at fault. These cases highlight the importance of understanding the roles and responsibilities of the physician and pharmacist in medication prescription and dispensation.

## INTRODUCTION

Physicians and pharmacists each have different roles and responsibilities in the process of safely prescribing and dispensing medications. Despite technology and systems designed to prevent harm, errors and oversights persist. The responsibility of an emergency physician to independently warn a patient of medication side effects is well established. A case that illustrates this duty is that of a woman with a history of migraines who had presented many times to the emergency department (ED) and received the same treatment regimen, which she had tolerated well previously. After receiving her usual treatment, however, she was discharged without being warned that the medicine could cause sedation. Shortly after discharge, she was involved in a motor vehicle accident that left her paraplegic. She litigated for “failure to warn” and was awarded $1.3 million.^1^

Our intention in this article is to capture the nuances involved when both a physician and a pharmacist are involved, such as when a medication is prescribed by a physician and dispensed by a pharmacist. Although the cases we discuss below are not specific to emergency medicine (EM), the principles apply to medication prescribing and dispensing in an EM setting.

### Case 1 - *Lovecchio v Rosenthal*

An 82-year-old man was discharged from the hospital after a cardiology admission. Upon discharge, the cardiologist prescribed outpatient amiodarone 1200 milligrams (mg) three times daily. A typical dose is 400 mg three times daily. The patient promptly filled the prescription. The pharmacist did not notice the excessive dose. At home, the patient took the first dose and four hours later suffered a cerebrovascular accident (CVA) that was later deemed by experts to be due to hypotension. He died 18 months later as a result of complications of the CVA. A lawsuit was filed claiming that the physician was in error for writing a prescription for 1200 mg three times daily and that the pharmacist should not have filled the prescription as written. A jury awarded a verdict of $1 million. The physician was responsible for $750,000, and the pharmacist was responsible for $250,000.2

### Case 2 - *Anonymous v Miller Pharmacy*

A 67-year-old cancer survivor picked up her regular prescription for methadone that was prescribed as 15 mg (three 5 mg tablets) twice daily. Four days later, she was found dead. It was discovered that she had been dispensed 10 mg tablets in error. The technician had typed the wrong dose and the pharmacist did not notice the error. The pharmacy and pharmacist settled for $325,000.3

### Case 3 - *Quick v Acaylar, Parnell, and Marlboro Drug Company*

A two-year-old female was prescribed ranitidine for gastrointestinal reflux by her pediatrician. After the pharmacist dispensed the medication at the prescribed dose, the child gradually developed tremors, shaking, ataxia, left eye deviation, and somnolence. Her parents litigated and claimed that their daughter was chronically overdosed with ranitidine. The usual dose was three-fourths of a teaspoon twice daily, and the child had been prescribed 3¼ teaspoons twice daily for 2–4 weeks. They claimed the pediatrician erred in prescribing that dose and that the pharmacist unsafely dispensed the medication. The pediatrician settled the case for a confidential amount. The pharmacist and pharmacy settled for $25,000.4

### Case 4 – *Anton v Brown*

A 37-year-old man was prescribed methadone for opioid addiction by his physician. The dose was 60 mg twice daily for 10 days followed by 30 mg twice daily for 10 days. The pharmacist called the physician to report there were no 60 mg tablets. The dose was changed to 1.5 tablets of 40 mg tablets twice daily. No future reduction in dose was discussed. Federal law at the time limited dosing to a maximum of 40 mg total daily. The patient developed nausea but was unable to reach his physician. A call to the pharmacist resulted in instructions to continue the medication until the physician was contacted. The patient was found dead the next day. Autopsy cited the drug interaction between escitalopram and methadone, which had been recently prescribed by the primary physician. The physician settled the lawsuit for $1 million, and the pharmacist contributed another $900,000 to the settlement.5

## DISCUSSION

### Adverse Drug Events

These four cases illustrate different sources of medication dosing errors, each of which lead to detrimental harm. Unfortunately, medication errors are not as rare as we would hope. Approximately 1 in 20 patients is exposed to preventable harm, and 25% of such incidents are medication-related.6 The landmark report *To Err is Human; Building a Safer Health System* by the Institute of Medicine estimated medication errors cause 1 of 131 outpatient and 1 of 854 inpatient deaths.7 In a study of adverse drug events in the ambulatory setting, the medication classes most frequently involved in adverse drug events were selective serotonin reuptake inhibitor (SSRI) agents (10%), beta-blockers (9%), angiotensin-converting-enzyme (ACE) inhibitors (8%), and nonsteroidal anti-inflammatory (NSAID) agents (8%).8

Traditionally, pharmacists have been viewed as protected from the duty to warn patients about their prescribed medications; this duty has historically rested upon the physician and drug companies. This legal concept will be discussed further below. However, some courts have recently ruled that pharmacists do have a duty to warn, particularly in cases in which there are known contraindications or clear errors in the prescription (for example, excessive dosing, as demonstrated in the cases above).9

A 2013 retrospective review of pharmacist liability insurance claims from 2002–2011 by Health Providers Service Organization (HPSO), a national provider of professional liability insurance for more than 70,000 pharmacists, demonstrated 162 closed pharmacist and pharmacy technician claims. A claim is considered closed when all of the following criteria occur: there is a medication-related incident; there is an adverse patient outcome; a claim is filed against the insured pharmacist or technician; and there is payout on behalf of the insured party. Individually insured pharmacists accounted for 93.8% of closed claims. Wrong drug (43.8%) and wrong dose (31.5%) claims together represented 75.3% of all the closed claims in the study sample. Failure to identify overdosing encompassed only 3.1% of all the closed claims in the analysis. However, the average paid overdose indemnity was substantially higher than the overall average paid indemnity. All claims except one in the “failure to identify overdose” category involved opioids.^10^

In a subsequent 2018 retrospective review sampling of HPSO pharmacist malpractice claims from 2012–2016, the annual number of closed claims more than doubled and the average total incurred payout per claim increased by 22.8%, to $124,407. While wrong drug and wrong dose errors continued to be the leading reasons for initiation of a claim, both decreased significantly relative to other causes with wrong drug claims accounting for 36.8% (from 43.8%) and wrong dose claims accounting for 15.3% (from 31.5%). These relative decreases are a result of large increases in other categories of allegations.^11^

Although the specific percentage of “failure to identify overdosing” claims is not explicitly stated in the 2018 report, it occurred the “most infrequently of all closed claims in the analysis.” However, the average total incurred claim payout for such “failure to identify overdosing” claims was $544,600. This represents 167% more than the next highest category (compounding calculation and/or preparation error) and 337% that of the overall average payout of $124,407. Overdose also represented the leading cause of death in the sample, accounting for 73.7% of claims associated with medication-related patient death.^10^

### Medications and Interactions

To further explore and illustrate the complexity of even a single dosing error and interaction, we take a closer evaluation of Case 4, *Anton v Brown*. This case involves concerns for both an excessive initial dose of methadone and potential interaction between methadone and the patient’s ongoing escitalopram treatment.

### Methadone

Methadone is a μ-opioid receptor agonist and likely N-methyl-D-aspartate receptor antagonist that is approved by the United States Food and Drug Administration (FDA) for opioid detoxification and maintenance therapy of opioid use disorder. Opioid agonist treatment with either methadone or combination of buprenorphine and naloxone has been proven to safely and effectively suppress illicit opioid use and reduce the risk of death.^12^ Methadone requires careful initiation, dose titration, medication changes, and discontinuation due to its pharmacokinetic properties, which vary widely from patient to patient. Characteristics that make methadone particularly difficult to safely administer include a long and highly variable half-life of 24–40 hours, a tendency for the medication to accumulate during initial treatment, and a risk of hazardous medication interactions.^11^

Methadone is metabolized by cytochrome P450 isozymes. Therefore, cytochrome P450 inducers, such as antiretrovirals, rifampin, barbiturates, and phenytoin, can accelerate metabolism of the drug, leading to earlier withdrawal. Alternatively, cytochrome P450 inhibitors, such as fluconazole, ketoconazole, cimetidine, fluoxetine, paroxetine, ciprofloxacin, macrolide antibiotics, and grapefruit juice, can prevent methadone metabolism, causing higher methadone plasma concentrations than intended and increasing the risk of sedation and overdose.^11^ Methadone has two primary hazards: respiratory depression and QT prolongation, particularly at higher doses. For these reasons, methadone is frequently administered as part of close monitoring programs —often daily—to ensure appropriate dosing and response to treatment.^11^ Treatment is initiated when there are no signs of sedation or intoxication and the patient shows signs of withdrawal.

Methadone dosing of 20–30 mg (maximum dose 30 mg) is administered orally initially. In patients with a low expected tolerance (ie, have not taken opioids for more than five days), lower initial dosing is recommended. The patient is reassessed 2–4 hours after the first dose. If additional dosing is determined necessary, an additional 5–10 mg can be administered. The maximum recommended total daily dose on the first day of treatment is 40 mg. Over the first week, dosing is adjusted cautiously based upon control of withdrawal symptoms 2–4 hours after administration. Over time, maintenance therapy targets a dosage that prevents opioid withdrawal for 24 hours, generally 80–120 mg/day. During discontinuation of therapy, dosing is decreased slowly, by no more than 10% in 10- to 14-day intervals, to prevent withdrawal.^11^

### Escitalopram

Escitalopram is an SSRI and S-enantiomer of racemic citalopram. Escitalopram is approved by the FDA for the treatment of major depressive disorder. It functions by enhancing serotonergic activity in the central nervous system (CNS) as a result of its inhibition of serotonin (5-HT) reuptake in CNS neurons.^13^

Escitalopram is metabolized by cytochrome P450 isozymes.^14^ Although the specific isozymes differ from those used by methadone and neither methadone nor escitalopram are known cytochrome P450 inhibitors or inducers, the potential for unknown and unpredictable interactions does exist.

Numerous hazards of escitalopram have been reported, including serotonin syndrome, QT prolongation, and torsades de pointes. Furthermore, SSRIs as a class have been shown to increase the risk of suicidal thinking and behavior in children, adolescents, and young adults with major depressive disorder and other psychiatric disorders in short-term studies. However, in patients beyond age 24, short-term studies did not show an increase in the risk of suicidality with antidepressants compared with placebo. Furthermore, in patients 65 and older, there was a reduction in risk of suicidal thinking and behavior with antidepressants compared with placebo.

Escitalopram is contraindicated in patients with hypersensitivity to citalopram or escitalopram, as well as patients who received a monoamine oxidase inhibitor in the previous 14 days, as these can interact to cause serotonin syndrome. Escitalopram also has a long list of medication interactions. For instance, strong evidence supports contraindication with linezolid and major interactions with lithium, both due to increased risk of serotonin syndrome, and risk with concurrent NSAID therapy due to increased risk of bleeding.^12^ A major drug-drug interaction warning exists between escitalopram and methadone due to concern for increased risk of both serotonin syndrome and QT prolongation.^12^

### Duty and Responsibility when Prescribing and Dispensing Medication

Pharmacists and physicians have separate duties when medications are prescribed and dispensed. Two cases clearly describe these respective duties.

The duty to warn patients of medication side effects rests with the prescribing physician. In *Morgan v Wal-Mart Stores, Inc*., a 12-year-old boy was prescribed desipramine for attention deficit hyperactivity disorder. The prescribing physician testified that she had shown the patient’s mother common side effects of desipramine in the *Physicians’ Desk Reference*. Two years later, after multiple physician visits, the child died of hypereosinophilic syndrome, a rare but known complication of desipramine. The parents filed suit against Wal-Mart for negligence “by failing to properly warn intended users of the hazards and harms associated with the use of the product.” The court ruled that the prescribing physician was liable and that the pharmacist had no duty to warn of medication side effects.^15^

Multiple other state courts have agreed that it is the physician’s duty to warn of these potential side effects. In *Frye v Walgreen*, the court ruled that pharmacists are not obligated to warn of all potential medication side effects. Simply placing warning labels on medication bottles does not imply that a pharmacist is accepting shared liability for a *physician’s* duty to warn.^16^ This leads to a dilemma for physicians: “How can I logistically warn patients of every side effect for every medication I prescribe?” In the ED, this can be practically accomplished by delegating the duty to the patient to “read the package inserts of prescribed medications” when discussing and providing discharge instructions. It is imperative for physicians to consider these warnings carefully when they add new medications or change doses of existing medications.

Pharmacists, on the other hand, have a duty to safely fill prescriptions and can be held liable for adverse outcomes if a prescription that a reasonable pharmacist would deem to be unsafe is still filled and dispensed. In *Brooks v Wal-Mart Stores, Inc*., the court ruled against a pharmacist who had filled a prescription for an excessive dose of prednisone (80 mg four times daily). The dose was confirmed with the physician at the time. The patient subsequently developed Nocardia pneumonia and cerebral aspergillosis. He underwent numerous surgeries and hospitalizations, ultimately developing renal failure. Despite confirming the initial dose with the physician, the pharmacist was nevertheless held solely liable for the medication error. The result was an award to the patient for $2.5 million. Although the physician is responsible for warning of side effects, the pharmacist must “exercise his [or her] own judgement as to whether any dosage prescribed, even if confirmed by the prescriber, would be harmful” and has an obligation to not fill a prescription he or she deems harmful.^17^

## CONCLUSION

We have presented four medicolegal cases involving medication prescription and dispensation errors that led to patient harm and subsequent settlements or jury awards to patients. These cases each involved scenarios in which a medication was inappropriately prescribed and/or inappropriately filled. Heretofore, it may not have been obvious to emergency care providers whether the physician or pharmacist is at fault. These cases have highlighted the importance of understanding the roles and responsibilities of the prescribing physician and filling pharmacist in medication prescription and dispensation. Although these legal cases do not originate in the ED, the legal principles hold true for ED practices. The above legal cases have established the legal duties of the physician who must warn and the pharmacist who must safely dispense. In some situations, these liabilities may be shared as noted in the cases above.

**Take-home Points**

1. The physician has a duty to warn patients of side effects and interactions of medications.
2. The pharmacist has no duty to warn patients of side effects but does have a duty to safely fill and dispense prescribed medications, including ensuring that a prescribed dose is safe.
3. We recommend that a physician reduce liability by directing patients to read the packaging inserts of their prescribed medications.
4. “Wrong drug” and “wrong dose” claims are the most common medication errors leading to monetary awards among pharmacy malpractice claims.
5. “Failing to identify overdose” is associated with the largest monetary awards among pharmacy malpractice claims.
6. The most common medications involved in outpatient adverse drug events are SSRIs, beta-blockers, ACE inhibitors, and NSAIDS.
